# Suppression of Dopamine Neurons Mediates Reward

**DOI:** 10.1371/journal.pbio.1002586

**Published:** 2016-12-20

**Authors:** Nobuhiro Yamagata, Makoto Hiroi, Shu Kondo, Ayako Abe, Hiromu Tanimoto

**Affiliations:** 1 Tohoku University Graduate School of Life Sciences, Sendai, Japan; 2 The University of Tokyo, Institute of Molecular and Cellular Biosciences, Tokyo, Japan; 3 National Institute of Genetics, Mishima, Japan; Cold Spring Harbor Laboratory, UNITED STATES

## Abstract

Massive activation of dopamine neurons is critical for natural reward and drug abuse. In contrast, the significance of their spontaneous activity remains elusive. In *Drosophila melanogaster*, depolarization of the protocerebral anterior medial (PAM) cluster dopamine neurons en masse signals reward to the mushroom body (MB) and drives appetitive memory. Focusing on the functional heterogeneity of PAM cluster neurons, we identified that a single class of PAM neurons, PAM-γ3, mediates sugar reward by suppressing their own activity. PAM-γ3 is selectively required for appetitive olfactory learning, while activation of these neurons in turn induces aversive memory. Ongoing activity of PAM-γ3 gets suppressed upon sugar ingestion. Strikingly, transient inactivation of basal PAM-γ3 activity can substitute for reward and induces appetitive memory. Furthermore, we identified the satiety-signaling neuropeptide Allatostatin A (AstA) as a key mediator that conveys inhibitory input onto PAM-γ3. Our results suggest the significance of basal dopamine release in reward signaling and reveal a circuit mechanism for negative regulation.

## Author Summary

Dopamine neurons in the midbrain of mammals fire action potentials in response to rewarding stimuli, while punitive stimuli or omission of reward suppress their activity. Different signs in the activity of dopamine neurons thus can encode appetitive and aversive values; however, how these bidirectional activities directly relate to behavior has yet to be elucidated. In fruit flies *Drosophila, en masse* activation of dopaminergic neurons in the protocerebral anterior medial (PAM) cluster has been shown to signal reward. Here, we demonstrate that a specific sub-class of these dopaminergic neurons, called PAM-γ3, mediates both aversive and appetitive reinforcement through activation and suppression of their activity, respectively. Notably, transient inactivation of the basal activity of PAM-γ3 neurons substitutes for reward and induces appetitive memory formation. Interestingly, we found that Allatostatin A, a neuropeptide that signals satiety, conveys inhibitory input onto PAM-γ3 neurons. Our results highlight the bidirectional activity of defined dopaminergic neurons, which underlies encoding of behaviorally relevant appetitive and aversive values.

## Abstract 3

Transient suppression of a specific subset of dopamine neurons signals reward in the fruit fly *Drosophila*, suggesting that basal dopamine activity underlies behaviorally relevant valence coding.

## Introduction

Dopamine plays a pivotal role in a wide range of motivation and learning [1–4]. In the fruit fly *Drosophila melanogaster*, phasic neurotransmission from specific dopamine neuron subsets mediate the reinforcing properties of salient unconditioned stimuli in associative learning [5–14]. These dopamine neurons endow positive and negative predictive values to associated environmental cues, thereby modulating the fly’s subsequent response to the conditioned cues [5,6,11–13]. In the fly brain, the protocerebral anterior medial (PAM) cluster of dopamine neurons conveys reward signals to the mushroom body (MB) [7,8]. While PAM cluster neurons exist from the larva, their cellular organization in the adult is much more complex and functionally heterogeneous [7,15,16]. Distinct dopamine neurons in the adult PAM cluster, for example, mediate appetitive and aversive reinforcement [5–7] and respond to sugar reward differently [7,12,17].

Besides phasic neurotransmission, recent studies revealed that valence-coding dopamine neurons have basal activity with fluctuating Ca^2+^ transient at the presynaptic terminals in the MB [17–21]. Ongoing dopamine release has been shown to control state-dependent consolidation of associative memory [18–21]. Considering the functional heterogeneity of the adult PAM cluster neurons [7,12,17], excitation and inhibition of dopamine neurons may signal appetitive and aversive values to drive bidirectional associative memories.

By characterizing PAM-γ3, a single class of dopamine neurons projecting to the γ3 region of the MB, we here show that sugar ingestion drives appetitive memory by suppressing the baseline activity of PAM-γ3. Furthermore, we searched for feeding-related signal molecules that inhibit PAM-γ3 and identified the neurons expressing the neuropeptide Allatostatin A (AstA). These results point to the importance of basal dopamine release and its negative regulation in reward processing.

## Results

**Fig 1:**
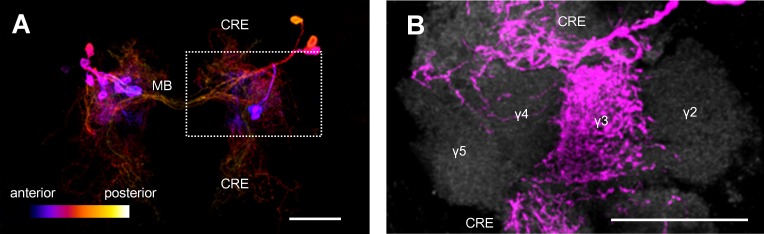
PAM-γ3 neurons specifically innervate the γ3 domain of the MB. (A) *MB441B-GAL4* specifically labels PAM-γ3 in the brain. (B) Magnification of the inset in A. The γ lobe is visualized with n-Cadherin (gray). CRE: crepine. Scale bars, 20 μm.

PAM cluster dopamine neurons are heterogeneous both morphologically and functionally and project to the distinct domains of the MB [7,12,17,22,23]. PAM-γ3 extends their dendritic arbor in the brain area surrounding the MB medial lobes (crepine) and projects specifically to the γ3 compartment of the MB (Fig 1A and 1B). While the majority of PAM neurons convey reward, previous studies implied that PAM-γ3 mediates aversive reinforcement [6,12]. However, additional GAL4 expression in other PAM neurons and nondopaminergic neurons of the driver line precluded identifying the responsible cells. We thus employed recently established split-GAL4 drivers *MB441B-GAL4* and *MB195B-GAL4* to specifically label 9 and 5 PAM-γ3 neurons, respectively [22] (Fig 1A).

**Fig 2:**
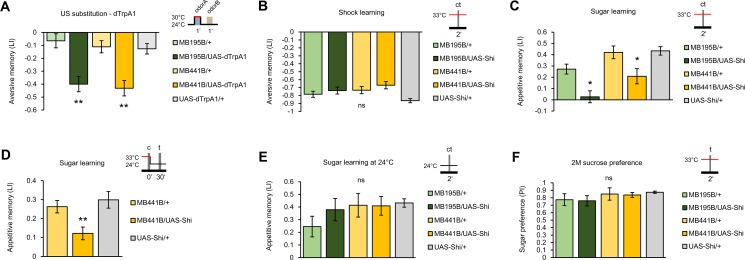
PAM-γ3 neurons are involved in acquisition of both aversive and appetitive memories. (A) Thermoactivation with *MB441B-GAL4* and *MB195B-GAL4* paired with an odor induces aversive memory of the paired odor. LI, learning index; *n* = 7–10. (B) Blockade of the PAM-γ3 neurons in *MB441B-GAL4*/*UAS-shi*^*ts1*^ and *MB195B-GAL4*/*UAS-shi*^*ts1*^ flies leaves electric shock–reinforced immediate aversive memory intact. *n* = 8. (C) In contrast, the same blockade impairs sugar-rewarded immediate appetitive memory. *n* = 12–20. These data are reproduced with the same genotypes, but outcrossed with a wild-type strain for four generations (S2 Fig). (D) Transient blockade of PAM-γ3 during training impairs acquisition of appetitive memory. *n* = 23–35. (E) The performance of *MB441B-GAL4/UAS-shi*^*ts1*^ *and MB195B-GAL4/UAS-shi*^*ts1*^ flies is normal at a permissive temperature (24°C, 30 min memory). *n* = 8. (F) Blockade of PAM-γ3 with *MB441B-GAL4/UAS-shi*^*ts1*^ or *MB195B-GAL4/UAS-shi*^*ts1*^ does not impair innate sucrose preference. PI, performance index; *n* = 6–8. c, conditioning; t, test. Bars and error bars indicate means and standard error of the mean (SEM), respectively, throughout this study. The numerical data used in all figures are included in S1 Data. * *p* < 0.05, ** *p* < 0.01; ns, not significant.

To examine the role of PAM-γ3 neurons in learning, we activated them by directing the expression of *dTrpA1*, a temperature-sensitive cation channel [24], using *MB441B-GAL4* and *MB195B-GAL4*. Simultaneous presentation of an odor with thermoactivation of the PAM-γ3 neurons induced robust conditioned avoidance of the paired odor (Fig 2A). Aversive memory established by thermoactivation of PAM-γ3 suggests their role in aversive reinforcement. As electric shock, compared to other aversive reinforcers, recruits the broadest set of dopamine neurons [6,10,14], we examined the requirement of the PAM-γ3 neurons in shock-induced aversive olfactory memory using targeted *shibire*^*ts1*^ (*shi*^*ts1*^) expression, a temperature-sensitive dominant negative form of dynamin GFPase that inhibits vesicle endocytosis [25]. Surprisingly, thermal blockade of PAM-γ3 using *MB441B* and *MB195B* did not significantly impair electric shock–reinforced aversive memory (Fig 2B). The result was same with the Shi^ts1^ blockade by another driver, *R58E02-GAL4*, which strongly drives transgene expression in the majority of the PAM cluster neurons, including PAM-γ3 [7] (S1 Fig).

As multiple classes of adult PAM neurons contribute to reward signaling differently [7,12,17], we examined the requirement of PAM-γ3 for sugar-induced appetitive memory. Contrary to aversive memory induced by depolarizing the PAM-γ3 neurons, thermal blockade of PAM-γ3 with *MB441B-GAL4/UAS-shi*^*ts1*^ and *MB195B-GAL4/UAS-shi*^*ts1*^ flies significantly impaired appetitive memory (Fig 2C). Moreover, blocking PAM-γ3 only during the acquisition of appetitive memory revealed a similar impairment, suggesting the role of PAM-γ3 in reward processing (Fig 2D). Their memory performance at a permissive temperature was not significantly different from those of the genetic controls (Fig 2E). The blockade of PAM-γ3 did not impair innate sugar preference at the concentration used for the learning experiment or lower (Figs 2F and S3A). These results suggest the selective requirement of PAM-γ3 for mediating sugar reward.

**Fig 3:**
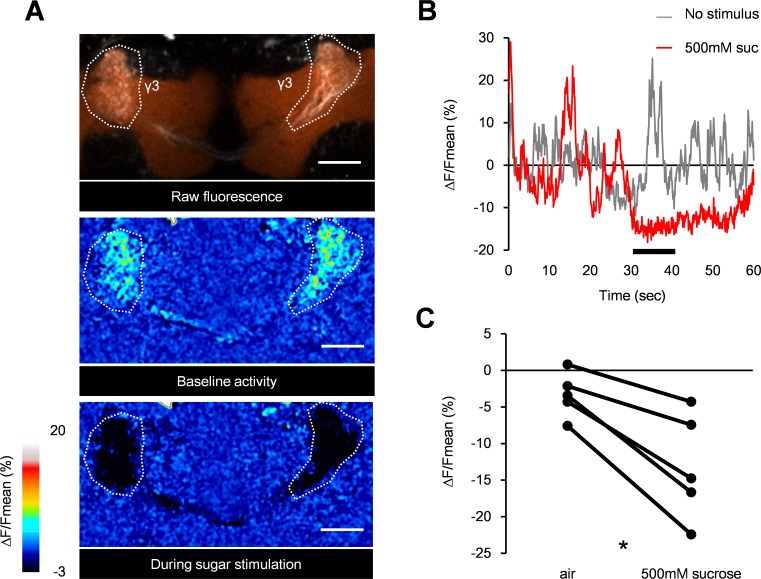
Sugar reward suppresses the baseline activity of PAM-γ3. (A) Representative raw fluorescence in the PAM-γ3 neurons of the *MB441B-GAL4*/*UAS-GCaMP5* fly with the mushroom bodies labelled with dsRed (orange). Two-photon images for one second before (baseline) and during the first second of sucrose stimulation (during sugar) are averaged and presented in a pseudocolor code. Region of interest is defined by terminals of PAM-γ3 in the medial lobes (outlined). Scale bar, 20 μm. (B) Time course of baseline activity (gray) and sugar response (red). Black bar: stimulus application. (C) Average calcium responses of PAM-γ3 neurons. Sucrose ingestion significantly reduces the activity level of PAM-γ3 (Wilcoxon matched-pairs signed rank test, *n* = 5). * *p* < 0.05.

As PAM-γ3 activation drives aversive memory [7], inhibition of the basal activity might be important for processing sugar reward. To examine this hypothesis, we imaged the Ca^2+^ response of PAM-γ3 by expressing GCaMP5 [26], a genetically encoded fluorescent calcium sensor, under the control of *MB441B-GAL4*. The baseline activity was fluctuating without stimulation (Fig 3A and 3B). Strikingly, sugar ingestion immediately silenced the baseline activity (Fig 3A, 3B and 3C). The Ca^2+^ level of PAM-γ3 neurons remained suppressed even after the ingestion (Fig 3B), which recovered to the baseline level approximately 20 s after the stimulus offset (Fig 3B). These data are compatible with the idea that PAM-γ3 neurons mediate appetitive reinforcement by acutely suppressing their baseline activity.

**Fig 4:**
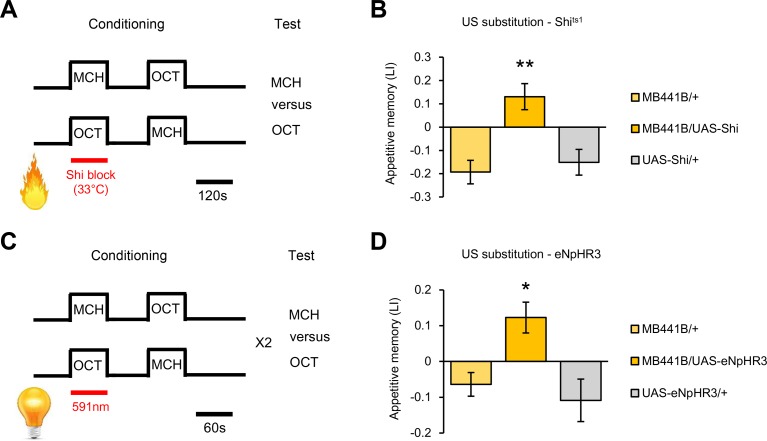
Transient inactivation of PAM-γ3 mediates reward. (A) Schematic diagram of reinforcement substitution experiment by transient inactivation with Shi^ts1^. (B) Paired suppression of PAM-γ3 activity using *MB441B-GAL4* induces significant appetitive memory. *n* = 11–19. (C) Schematic diagram of reinforcement substitution experiment by light-induced inactivation of PAM-γ3 with eNpHR3.0. (D) Paired suppression of PAM-γ3 activity with eNpHR3.0 using *MB441B-GAL4* induces significant appetitive memory. *n* = 8–11. * *p* < 0.05, ** *p* < 0.01.

We hypothesized that transient inactivation of the PAM-γ3 dopamine neurons may be sufficient to signal appetitive reinforcement. Similar to the reinforcement substitution experiment with *dTrpA1*-mediated depolarization (Fig 1), we paired the Shi^ts1^ blockade of PAM-γ3 with one of the two odors; temperature was shifted only during the presentation of the conditioned odor (Fig 4A). This protocol is different from the former Shi ^ts1^ experiments (Fig 2C and 2D), in which PAM-γ3 was blocked during both CS^+^ and CS^-^. The paired blockade of PAM-γ3 in *MB441B-GAL4/UAS-shi*^*ts1*^ flies indeed induced significant appetitive odor memory (Fig 4B). To confirm this appetitive memory, we established an optogenetic silencing approach by using engineered halorhodopsin (eNpHR) [27], a light-gated chloride ion pump. Transient blockade of the PAM-γ3 output by applying yellow light (591 nm) during the odor presentation resulted in an induction of appetitive memory (Fig 4C and 4D). We thus conclude that the suppression of PAM-γ3 baseline activity is sufficient to signal appetitive reinforcement.

**Fig 5:**
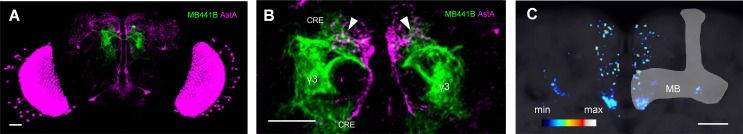
Allatostatin A neurons project to PAM-γ3. (A) Expression patterns of *AstA-* (magenta) and *MB441B-GAL4* (green) neurons aligned in a standard brain. Three brains for each line were examined, which essentially exhibited the same overlap pattern. (B) Magnification of (A) around the MB γ lobe. These processes overlap in the crepine (CRE; arrowheads). (C) Complementary GFP moieties are expressed in the AstA and PAM-γ3 neurons. The GRASP signals from six animals are averaged and pseudocolor-coded. Gray shades indicate the MB. Scale bar, 20 μm.

How does sugar ingestion suppress PAM-γ3 activity? Many neuropeptides are known to reflect feeding states and inhibit target cells through their receptors coupled with inhibitory G proteins [28]. We thus examined the expression patterns of a series of neuropeptide-related GAL4 drivers for their potential connection with the PAM-γ3 dendrites in silico [12]. Image registration of confocal stacks of the expression of neuropeptide GAL4 lines and *MB441B-GAL4* into a standard brain revealed a spatial overlap between the processes of *AstA* and the PAM-γ3 neurons (Fig 5A and 5B). This putative connection was experimentally confirmed using reconstituted GFP signal between PAM-γ3 and AstA neurons by the GFP reconstitution across synaptic partners (GRASP) technique [29] (Fig 5C).

**Fig 6:**
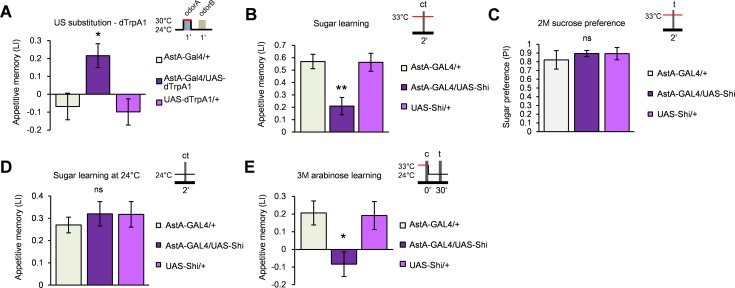
Allatostatin A neurons mediate reward. (A) Thermoactivation of *AstA*-expressing neurons using *UAS-dTrpA1* induces immediate appetitive memory. *n* = 16. (B–C) Blockade of the *AstA* neurons with *UAS-shi*^*ts1*^ impairs immediate appetitive memory (B, *n* = 8) while leaving sucrose preference intact (C, *n* = 6–8). (D) Normal learning performance of *AstA-GAL4/UAS-Shi*^*ts1*^ flies at a permissive temperature. *n* = 6–8. (E) Blockade of the *AstA* neurons with *UAS-shi*^*ts1*^ during learning impairs immediate appetitive memory of nonnutritive sugar arabinose. *n* = 9–16. ns, not significant, * *p* < 0.05, ** *p* < 0.01.

AstA was shown to signal satiation in *Drosophila* [30] and inhibits target neurons [31]. We therefore asked the involvement of the AstA neurons in mediating sugar reward. Thermoactivation of *dTrpA1* with *AstA-GAL4*, which exclusively labels a subset of AstA immunopositive neurons [30], resulted in the formation of appetitive odor memory (Fig 6A). The blockade of AstA neurons during learning significantly lowered appetitive memory of both sucrose (Fig 6B) and nonnutritive sugar arabinose (Fig 6E). The sugar preference of *AstA-GAL4/UAS-shi*^*ts1*^ flies and their memory performance at a permissive temperature were unimpaired (Figs 6C and 6D and S3B). Thus, AstA-expressing neurons are necessary and sufficient for mediating the reinforcement property of sugar reward, likely sweetness.

**Fig 7:**
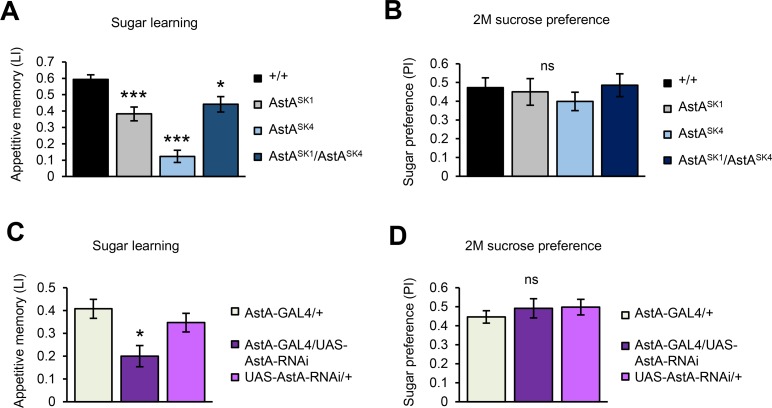
AstA protein is required for sugar learning but dispensable for innate sucrose preference. (A) AstA mutant flies (*AstA*^*SK1*^, *AstA*^*SK4*^, and *AstA*^*SK1/SK4*^) are defective in sugar memory performances. *n* = 10–32. (B) Sucrose preference of *AstA* mutant flies are indistinguishable from that of the wild-type strain. *n* = 8–16. (C-D) *AstA* knockdown using *AstA-GAL4* impairs sugar memory (C, *n* = 12–16), while leaving sucrose preference intact (D, *n* = 8). ns, not significant, * *p* < 0.05, *** *p* < 0.001.

To confirm that the AstA protein is the underlying modulatory signal, we generated multiple null alleles of *AstA* using the CRISPR/Cas9 system. Appetitive memory of these mutant flies was significantly impaired (Fig 7A) while leaving their innate sugar preference unaffected (Fig 7B). Moreover, we also generated an RNA interference (RNAi) fly line against AstA based on the small hairpin RNA (shRNA) technique [32]. Down-regulation of *AstA* using *AstA-GAL4* resulted in an impaired appetitive learning (Fig 7C) while leaving sugar preference intact (Fig 7D). Given that AstA is an inhibitory neuropeptide [31], these results suggest that the AstA release conveys sugar reward by inhibiting the PAM-γ3 dopamine neurons.

**Fig 8:**
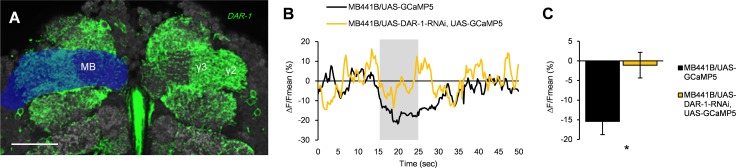
DAR-1 signaling is required for sugar-induced suppression of PAM-γ3 activity. (A) *DAR-1-GAL4* expression in PAM-γ3. The MB γ lobe is labelled in blue. Scale bar, 20 μm. (B) Averaged time course of the baseline activity of PAM-γ3 with (yellow, *n* = 6 animals) or without (black, *n* = 5) *DAR-1* knockdown. Gray bar: 500 mM sucrose application for 10 s. (C) Averaged calcium responses of PAM-γ3 neurons. *DAR-1* knockdown significantly attenuated sugar-induced suppression of PAM-γ3 activity (Mann–Whitney test, *n* = 5–6). * *p* < 0.05.

In order to visualize the distribution of an AstA receptor, we inserted the *GAL4* transgene into the C-terminus of the *Allatostatin A receptor 1* (*DAR-1*) coding region [33] by means of the CRISPR-Cas9 system. Confocal examination of *DAR-1-GAL4* expression revealed positive labelling in the dopamine neurons projecting to the MB, including PAM-γ3 (Figs 8A and S4).

If AstA/DAR-1 signaling also works in an inhibitory manner in the PAM-γ3, down-regulation of *DAR-1* may weaken the PAM-γ3 suppression. To test this, we generated RNAi fly strains against *DAR-1* based on the shRNA technique. Strikingly, knocking down *DAR-1* in the PAM-γ3 significantly attenuated the suppression of the baseline activity (Fig 8B and 8C). Altogether, we propose that AstA provides an inhibitory signal to PAM-γ3 upon the ingestion of rewarding substances.

**Fig 9:**
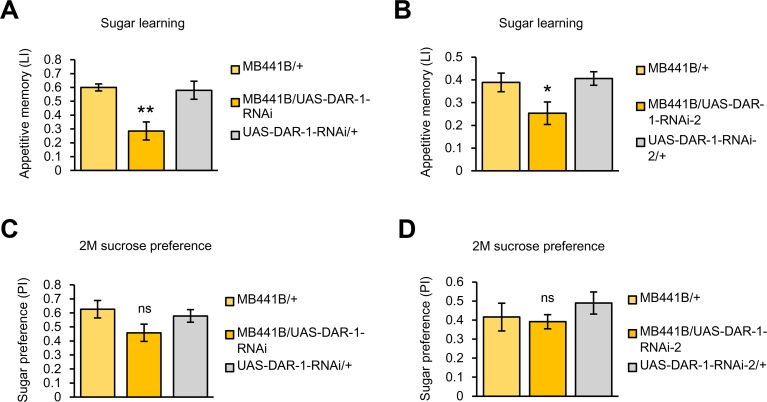
Requirement of *DAR-1* in the PAM-γ3 in sugar learning. (A) Down-regulation of *DAR-1* expression in PAM-γ3 by *MB441B* impairs immediate sugar memory. *n* = 12–15. (B) Knocking down *DAR-1* in *MB441B-GAL4* driven by second line of *UAS-DAR-1-RNAi* impairs sugar memory. *n* = 24–26. (C) Knocking-down *DAR-1* in the PAM-γ3 driven by *MB441B-GAL4* did not alter innate sugar preference. *n* = 9. (D) Knocking down *DAR-1* in the *MB441B-GAL4* driven by second line of *UAS-DAR-1-RNAi* did not alter innate sucrose preference. *n* = 8. ns, not significant. * *p* < 0.05, ** *p* < 0.01.

To examine the behavioral effect of AstA/DAR-1 signaling in the PAM-γ3, we down-regulated *DAR-1* expression in the PAM-γ3 neurons and examined their appetitive memory. Consistent with our proposal, the knockdown significantly impaired sugar learning (Fig 9A and 9B) while leaving the innate sugar preference intact (Fig 9C and 9D).

**Fig 10:**
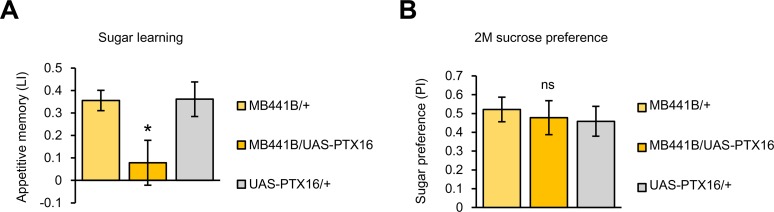
Requirement of Gαo signaling in PAM-γ3 for appetitive learning. (A) Pertussis toxin expression in the PAM-γ3 impairs immediate sugar memory. *n* = 8. (B) Innate sugar preference is not significantly impaired. *n* = 8. ns, not significant, * *p* < 0.05.

AstA/DAR-1 signaling was shown to suppress neuronal activity in receiving cells through Gαi/o signaling [31,34]. To examine the intracellular mechanism of AstA/DAR-1 signaling in PAM-γ3, we inhibited the Gαo subunit by expressing the pertussis toxin with *MB441-GAL4* [35]. As these flies had defective sugar memory (Fig 10A) but unimpaired sugar preference (Fig 10B), we suggest that DAR-1 inhibits PAM-γ3 activity by recruiting Gαo.

## Discussion

Sugar ingestion triggers multiple reward signals in the fly brain [7,12]. We here provided lines of evidence that part of the reward is signaled by inactivating dopamine neurons (Figs 1–4). The role of PAM-γ3 highlights the striking functional heterogeneity of PAM cluster dopamine neurons. The decrease and increase of dopamine can convey reward to the adjacent compartments of the same MB lobe—γ3 and γ4—(Figs 3 and 4) [9,17]. The reward signal by the transient decrease of dopamine is in stark contrast to the widely acknowledged role of dopamine [36,37]. Midbrain dopamine neurons in mammals were shown to be suppressed upon the presentation of aversive stimuli [38] or the omission of an expected reward, implying valence coding by the bidirectional activity [39]. As depolarization of PAM-γ3 can signal aversive reinforcement (Fig 2), these neurons convey the opposite modulatory signals to the specific MB domain by the sign of their activity. Intriguingly, the presentation and cessation of electric shock act as punishment and reward, respectively [40]. Such bidirectional activity of PAM-γ3 may represent the presentation and omission of reward (Figs 1–4).

While thermoactivation of PAM-γ3 induced robust aversive memory, blocking their synaptic transmission did not affect shock learning, leaving a question regarding their role in endogenous aversive memory process. PAM-γ3 may only be involved in processing aversive reinforcement different from electric shock—like heat [10] or bitter taste [11,41]—or respond only to the omission of a reward as pointed above [40,42]. However, two studies show that dopamine neurons mediating aversive reinforcement of high temperature and bitter *N*,*N*-Diethyl-3-methylbenzamide (DEET) are part of those for electric shock. Identification of such aversive stimuli that are signaled by PAM-γ3 activation is certainly interesting, as it is perceived as the opposite of sugar reward and thus provides the whole picture of the valence spectrum. Another scenario where sufficiency and necessity do not match is the compensation of the reinforcing effect by other dopamine cell types (e.g. MB-M3 [6]). The lack of PAM-γ3 requirements for electric shock memory may be explained by a similar mechanism.

How can the suppression of PAM-γ3 modulate the downstream cell and drive appetitive memory? Optogenetic activation of the MB output neurons from the γ3 compartment induces approach behavior [43]. This suggests that the suppression of the PAM-γ3 neurons upon reward leads to local potentiation of Kenyon cell output. This model is supported by recent studies showing the depression of MB output synapses during associative learning [17,44–46]. A likely molecular mechanism is the de-repression of inhibitory D2-like dopamine receptors, *DD2R* [47]. As D2R signaling is a widely conserved mechanism [48], it may be one of the most ancestral modes of neuromodulation.

Furthermore, recent anatomical and physiological studies demonstrated that different MB-projecting dopamine neurons are connected to each other and act in coordination to respond to sugar or shock [17, 43]. Therefore, memories induced by activation or inhibition of PAM-γ3 may well involve the activity of other dopamine cell types.

**Fig 11:**
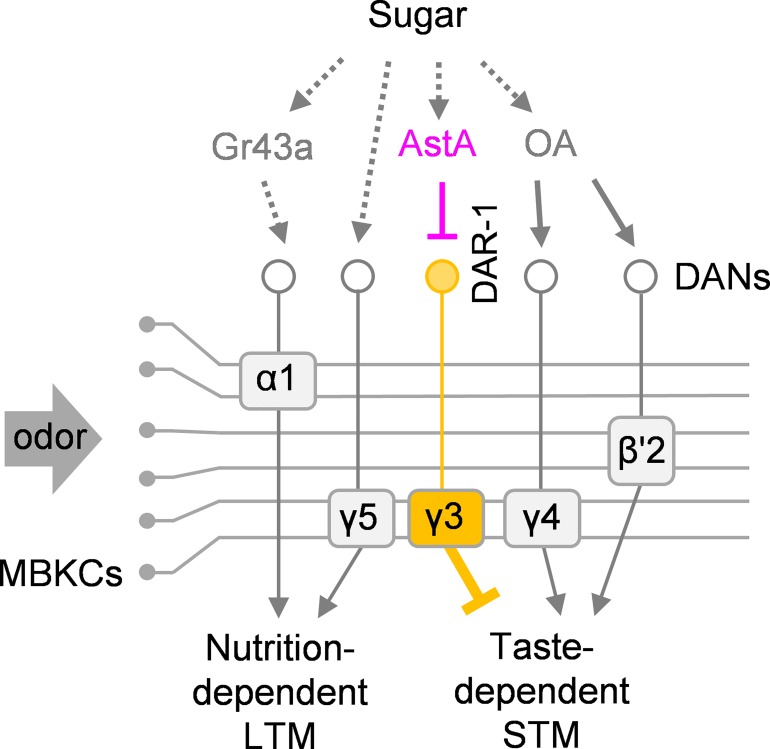
Reward signals by excitation and inhibition of dopamine neurons. Schematic diagram of the reward circuits in the MB. Sugar ingestion activates multiple modulatory pathways, such as octopamine and AstA, that bidirectionally regulate distinct dopamine neurons. These dopamine neurons convey reward signals to the MB by their activation or inhibition and induce appetitive memory.

Our finding that appetitive reinforcement is encoded by both activation and suppression of dopamine neurons raises the question as to the complexity of reward processing circuits (Fig 11). It is, however, reasonable to implement a component like PAM-γ3 as a target of the satiety-signaling inhibitory neuropeptide AstA. Intriguingly, the visualization of AstA receptor distribution by *DAR-1-GAL4* revealed expression in two types of MB-projecting dopamine neurons: PAM-γ3 and MB-MV1 (also named as PPL1- γ2α’1). Given the roles of MB-MV1 in aversive reinforcement and locomotion arrest [6,10,17,19], AstA/DAR-1 signaling may also inhibit a punishment pathway upon feeding. We thus speculate that this complex dopamine reward circuit may be configured to make use of bidirectional appetitive signals in the brain (Fig 11).

## Materials and Methods

### Flies

Canton-S was used as a wild-type strain. Generation and basic characterization of split-GAL4 drivers, *w;MB441B-GAL4* and *w;MB195B-GAL4*, is described in [22]. *w;;R58E02-GAL4* and *w;;AstA-GAL4* are described in [7,30]. *UAS-PTX16* is described in [35]. *AstA* mutants were generated as described previously [49]. Two null alleles, *AstA*^*SK1*^ and *AstA*^*SK4*^, in which the initiation methionine codon is deleted, were used throughout the present study. *DAR-1-GAL4* was generated by CRISPR/Cas9-mediated targeted integration of a *GAL4* transgene immediately in front of the stop codon of the *DAR-1* gene. The *GAL4* transgene contains a T2A peptide at the amino-terminus [50], such that GAL4 protein is synthesized from the same transcript as *DAR-1* through the ribosomal skipping mechanism. The insertion was validated with genomic PCR and sequencing. Flies were raised at 24°C except for DAR-1 and AstA knockdown experiments. Thermoactivation experiments (Fig 1A) used the F1 progeny of the crosses between females of *w;UAS-dTrpA1* [24] or *w* and males of the *GAL4* drivers. Synaptic blockade experiments (Figs 2, 4, 6, S1, S2 and S3) used the F1 progeny of the crosses between females of *w;;UAS-shi*^*ts1*^ [51] or *w* and males of the *GAL4* drivers. For the *AstA* (Fig 7) and *DAR-1* knockdown experiments (Figs 8 and 9), we generated a *UAS-AstA-RNAi* and two *UAS-DAR-1-RNAi* transgenes in the Vailum20 vector [52]. The 21-bp, gene-specific sequence was: ACGCAGCGACTACGACTACGA (AstA), TCGGTCATTATTCAGATTATA (DAR-1), and CACGATAGGGATCTCTGTCAA (DAR-1), respectively. Females of those flies were crossed with males of the *GAL4* drivers or *w*. The F1 progeny was raised at 24°C until 6 d after hatch, moved to 30°C, and kept for 1–14 d before experiments. For immunohistochemistry, a female reporter strain *y w UAS-mCD8*::*GFP;UAS-mCD8*::*GFP;UAS-mCD8*::*GFP* or *w;58E02-LexA LexAop-mCD4*::*GFP11;UAS-mCD4*::*GFP1-10* was crossed to male *GAL4* drivers, *w;MB441B-GAL4*, *w;;AstA-GAL4*, *w;;DAR-1-GAL4*. Flies used for whole-mount immunohistochemistry were aged to 5–10 d after eclosion. For Ca^2+^ imaging experiments, males of *w; mb247-dsRed*, *UAS-GCaMP5; UAS-GCaMP5* [26] or *w; UAS-V20-DAR-1-RNAi; UAS-GCaMP5* were crossed to *w;MB441B-GAL4* females and raised at 24°C (Figs 3 and 8). For detailed fly genotypes used for behavioral experiments, see S1 Table.

### Immunohistochemistry

Immunolabelling for the analysis of *GAL4* lines was performed as previously described [5–7,12]. Either 97% TDE [53] or Vectashield (VECTASHIELD®, Vector) was used as mounding medium. The employed primary antibodies were the rabbit anti-GFP (1:1000; Invitrogen; A11122) or rat anti-N-cadherin (DN-EX #8; 1:100; Developmental Studies Hybridoma Bank). The employed secondary antibodies were the cross-adsorbed secondary antibodies to IgG (H+L): AlexaFluor-488 goat anti-rabbit (1:1000; Invitrogen; A11034) or Cy3 goat anti-rat (1:200; Jackson Labs). Optical sections of whole-mount brains were sampled with a confocal microscope (Olympus FV1200). Confocal stacks were analyzed with the open-source software Image-J (National Institute of Health) and Fiji [54].

### Image Registration

Landmark matching–based affine and nonrigid registration of whole brains was performed as previously described [12]. Confocal images of entire brains of *GAL4/UAS-mCD8*::*GFP* flies were scanned with *n-cadherin* (*n-Cad*) counterstaining and registered into the standardized brain by referring the *n-Cad* channel. The transformations computed with the *n-Cad* channel were then applied to the *mCD8*::*GFP* channel. The registered images were assigned into the standardized brain and represented as different colors using ImageJ.

### Behavioral Assays

The conditioning and testing protocol was as described previously [7,12]. Briefly, for sugar learning and the US substitution experiment by *dTrpA1*-mediated thermoactivation, a group of approximately 50 flies in a training tube alternately received octan-3-ol (OCT; Merck) and 4-methylcyclohexanol (MCH; Sigma-Aldrich) for 1 min in a constant air stream with or without dried sucrose paper or 30°C heat. For the US substitution experiment by *Shi*^*ts1*^-mediated thermoinactivation (Fig 4), a group of approximately 50 flies in a training tube alternately received OCT and MCH for 2 min in a constant air stream with or without 33°C heat. For the US substitution experiment by *eNpHR3*-mediated light-inactivation (Fig 4), a group of approximately 50 flies were put into a custom-made LED-embedded aluminum tube and alternately received OCT and MCH for 1 min twice in a constant air stream with or without a continuous light exposure (591 nm). The light intensity was approximately100 mW/mm^2^ at a distance of 10 mm from the LED, measured with the Laser Power Meter Console (Thorlabs, PM100A). Flies were fed with all-trans-retinal contained food (2.5 mM) at least for 3 d before the experiments. OCT and MCH were diluted 10% in paraffin oil (Sigma-Aldrich) and placed in a cup with a diameter of 3 mm or 5 mm, respectively. After a given retention time, the conditioned response of the trained flies was measured with a choice between CS+ and CS- for 2 min in a T maze. The memories were tested immediately after training unless otherwise stated. The restrictive temperature for the experiments with *UAS-shi*^*ts1*^ was 33°C and the permissive temperature was 24°C, measured with the VC-960 digital multimeter (Voltcraft). For memory retention, trained flies were kept in a vial with moistened filter paper. After a given retention time, the trained flies were allowed to choose between MCH and OCT for 2 min in a T maze. A learning index was then calculated by taking the mean preference of the two reciprocally trained groups. Half of the trained groups received reinforcement together with the first presented odor and the other half with the second odor to cancel the effect of the order of reinforcement.

### Statistics

Statistical analyses were performed with Prism5 (GraphPad). Most of the data did not violate the assumption of normal distribution and homogeneity of variance. Therefore, the data were analyzed with parametric statistics: one-way analysis of variance followed by the planned pairwise multiple comparisons (Bonferroni two-tailed test). Figs 2B, 2F, 6C, 6D, 7B, 9A, S1 and S2 were analyzed with nonparametric statistics: Kruskal–Wallis one-way analysis of variance followed by the planned pairwise multiple comparisons (Dunn’s test). The significance level of statistical tests was set to 0.05. For detailed results for statistical tests, see S1 Table. The numerical data used in all figures are included in S1 Data.

### In Vivo Calcium Imaging

The expression of *GCaMP5* [26] calcium reporter was targeted to PAM-γ3 neurons by crossing *MB441B-GAL4* to *mb247-dsRed*, *UAS-GCaMP5*, or *UAS-V20-DAR-1-RNAi; UAS-GCaMP5* flies. Flies that were 2 to 3 d old from the offspring were starved at 25°C for 24 h on a Kimwipe soaked with water. For DAR-1 knockdown experiments, flies were aged to 8–12 d after eclosion. Flies were then prepared for in vivo imaging by confocal microscopy as previously described [55]. Fluorescence was recorded in a transverse section of the brain. Recordings were made with a frame rate of 2 Hz in two animals, 10 Hz in two animals, and for the rest at 5 Hz, which did not alter the results. Each fly was presented with a droplet of 500 mM sucrose. The fly had access to the gustatory stimulus for 10 s. Image analysis was performed essentially as described previously [55]. Briefly, an object in each recording was stabilized by phase correlation–based image alignment using dsRed signal, then GCaMP5 signal was used as a fluorescent F value. In each animal, a region of interest in the left hemisphere was used. The baseline value of fluorescence F_mean_ was calculated as the average of ΔF/F_mean_ over 15 s before the start of the stimulation.

## Supporting Information

S1 Fig Neurotransmission from the PAM neurons is dispensable for shock learning. Blockade of the PAM neurons in *R58E02-GAL4*/*UAS-shi*^*ts1*^ flies did not impair shock learning. *n* = 16. Results are means ± SEM. ns, not significant.
(TIF)

S2 Fig Shibire blockade with back-crossed MB441B impairs appetitive memory. *n* = 8–10. Results are means ± SEM. * *p* < 0.05.
(TIF)

S3 Fig Low concentration sugar preference of *MB441B-GAL4/UAS-shi*^*ts1*^ and *AstA-GAL4/UAS-shi*^*ts1*^ flies. (A) Blockade of the PAM-γ3 neurons in *MB441B-GAL4/UAS-shi*^*ts1*^ flies (A, *n* = 8) or AstA neurons in *AstA-GAL4/UAS-shi*^*ts1*^ flies (B, *n* = 8) did not impair 500 mM sucrose preference. Results are means ± SEM. ns, not significant.
(TIF)

S4 Fig DAR-1 expression in the brain revealed by knockin *DAR-1-GAL4*. Scale bar, 20 μm.
(TIF)

S1 Table List of crosses and statistics for behavior experiments (DOCX)

S1 Data Excel spreadsheet containing, in separate sheets, the underlying numerical data and statistical analysis for Fig panels 2A, 2B, 2C, 2D, 2E, 2F, 3C, 4B, 4D, 6A, 6B, 6C, 6D, 6E, 7A, 7B, 7C, 7D, 8C, 9A, 9B, 9C, 9D, 10A and 10B, S1, S2, S3A and S3B. (XLSX)

## Abbreviations

AstA: Allatostatin A
MB: mushroom body
PAM: protocerebral anterior medial
GRASP: GFP reconstitution across synaptic partners
RNAi: RNA interference
shRNA: small hairpin RNA
