# Supplementary figures and images for: Suppression of Dopamine Neurons Mediates Reward
Source: PLoS Biol. 2016 Dec 20;14(12):e1002586. doi: 10.1371/journal.pbio.1002586 (PMC5172549; doi:10.1371/journal.pbio.1002586)

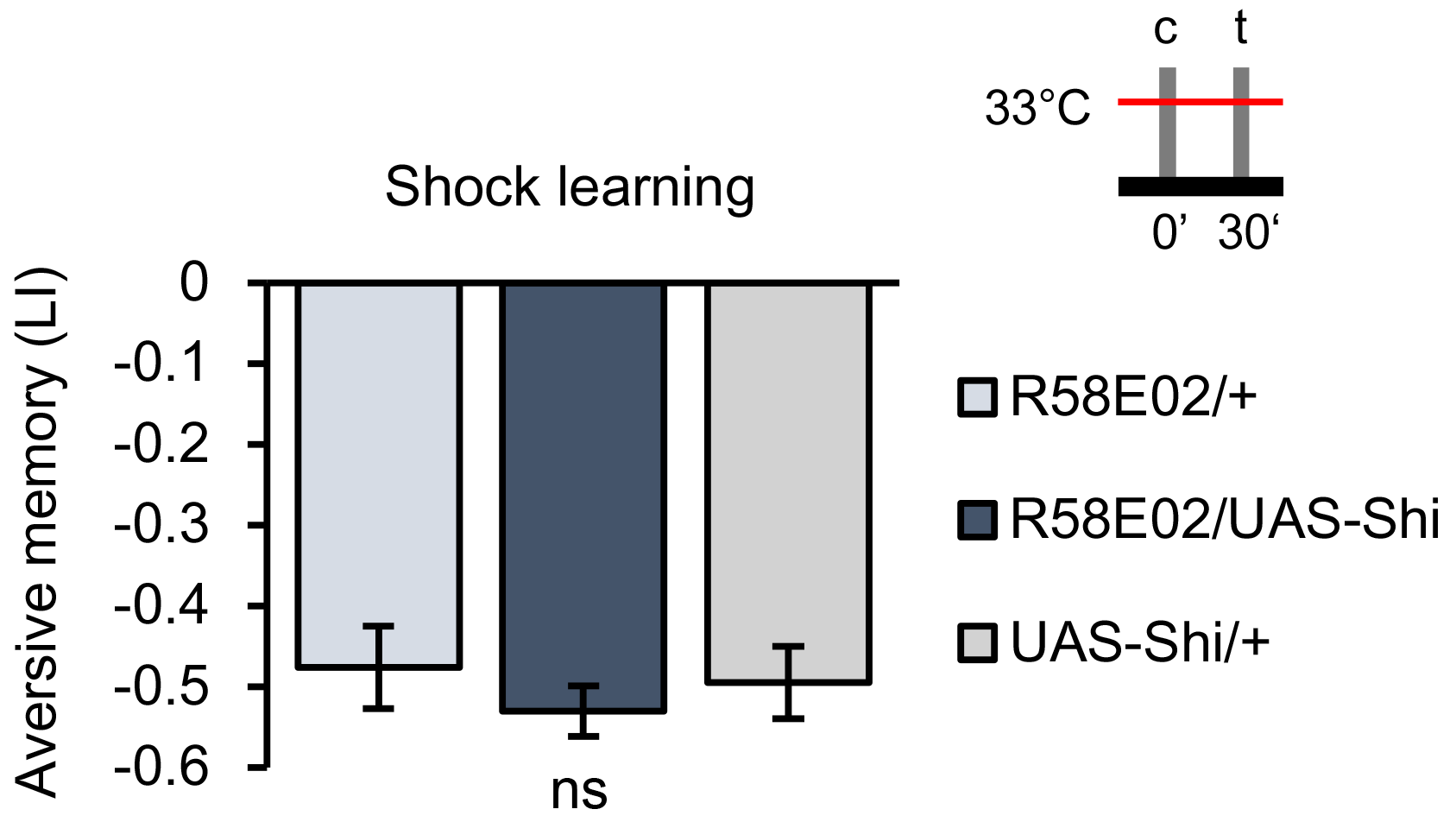

Supplement: S1 Fig — Blockade of the PAM neurons in R58E02-GAL4/UAS-shits1 flies did not impair shock learning. n = 16. Results are means ± SEM. ns, not significant. (TIF) [file pbio.1002586.s001.tif]

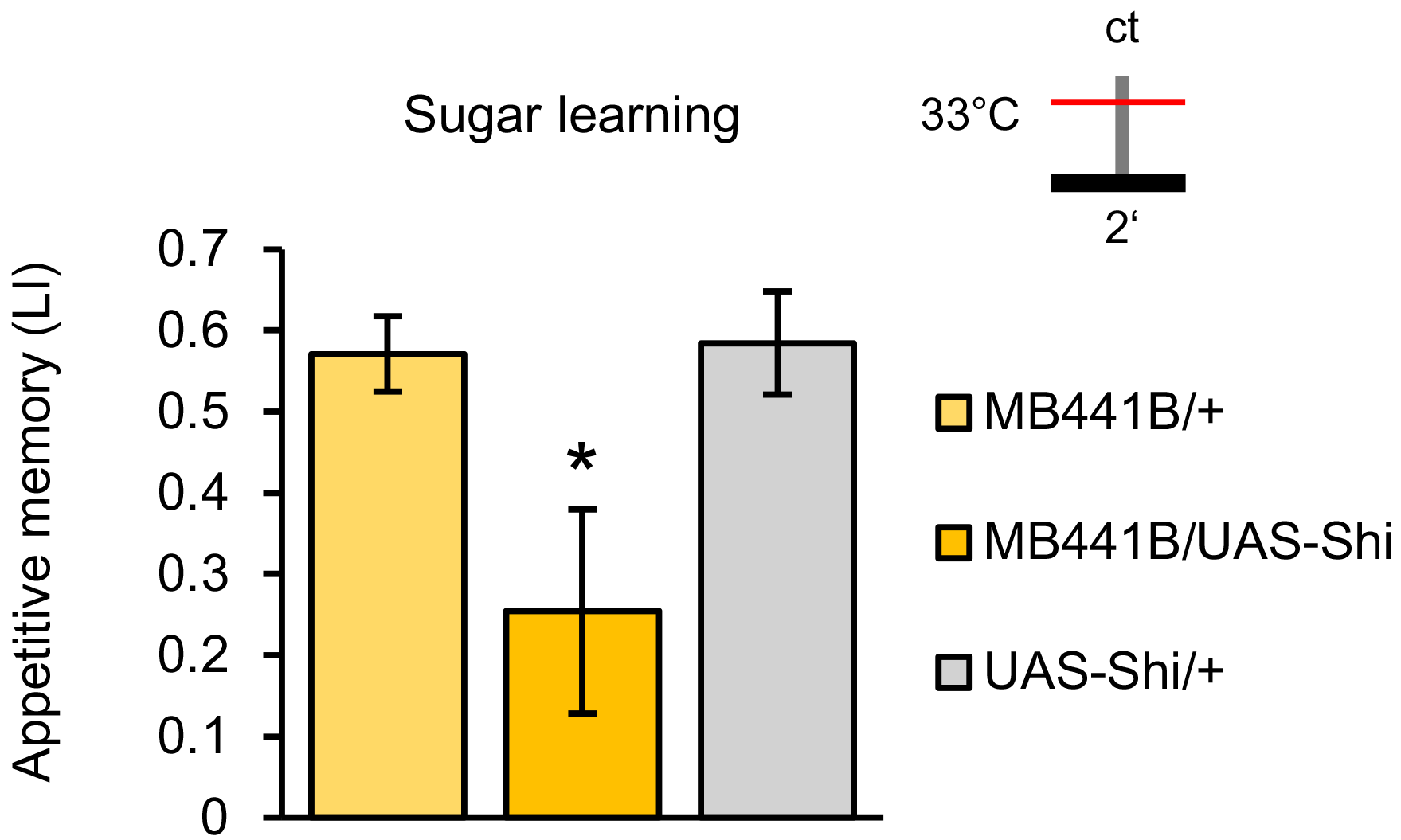

Supplement: S2 Fig — n = 8–10. Results are means ± SEM. * p < 0.05. (TIF) [file pbio.1002586.s002.tif]

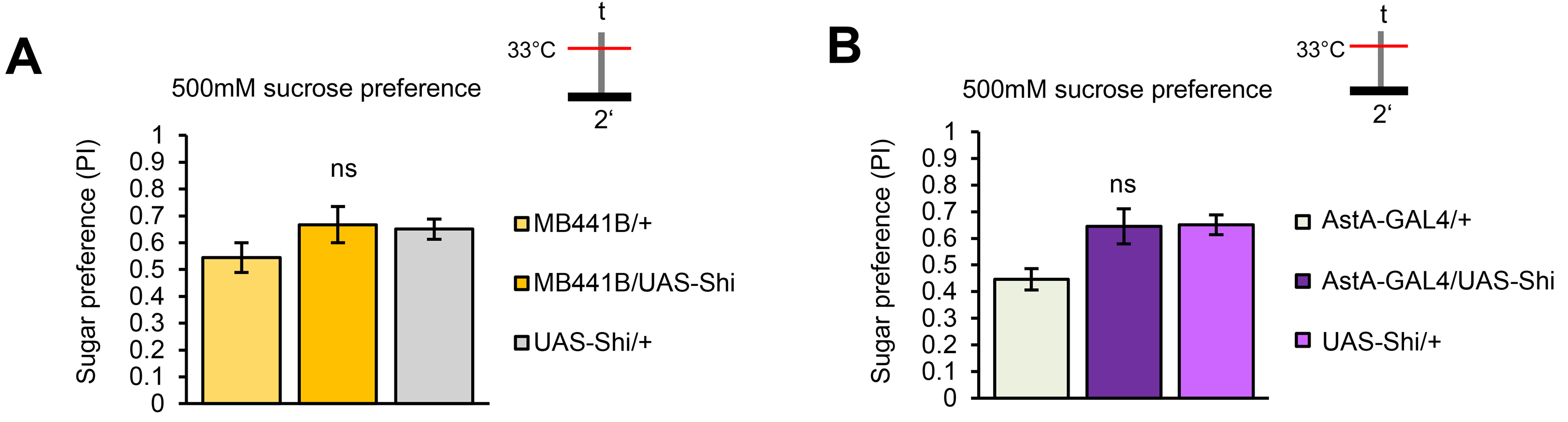

Supplement: S3 Fig — (A) Blockade of the PAM-γ3 neurons in MB441B-GAL4/UAS-shits1 flies (A, n = 8) or AstA neurons in AstA-GAL4/UAS-shits1 flies (B, n = 8) did not impair 500 mM sucrose preference. Results are means ± SEM. ns, not significant. (TIF) [file pbio.1002586.s003.tif]

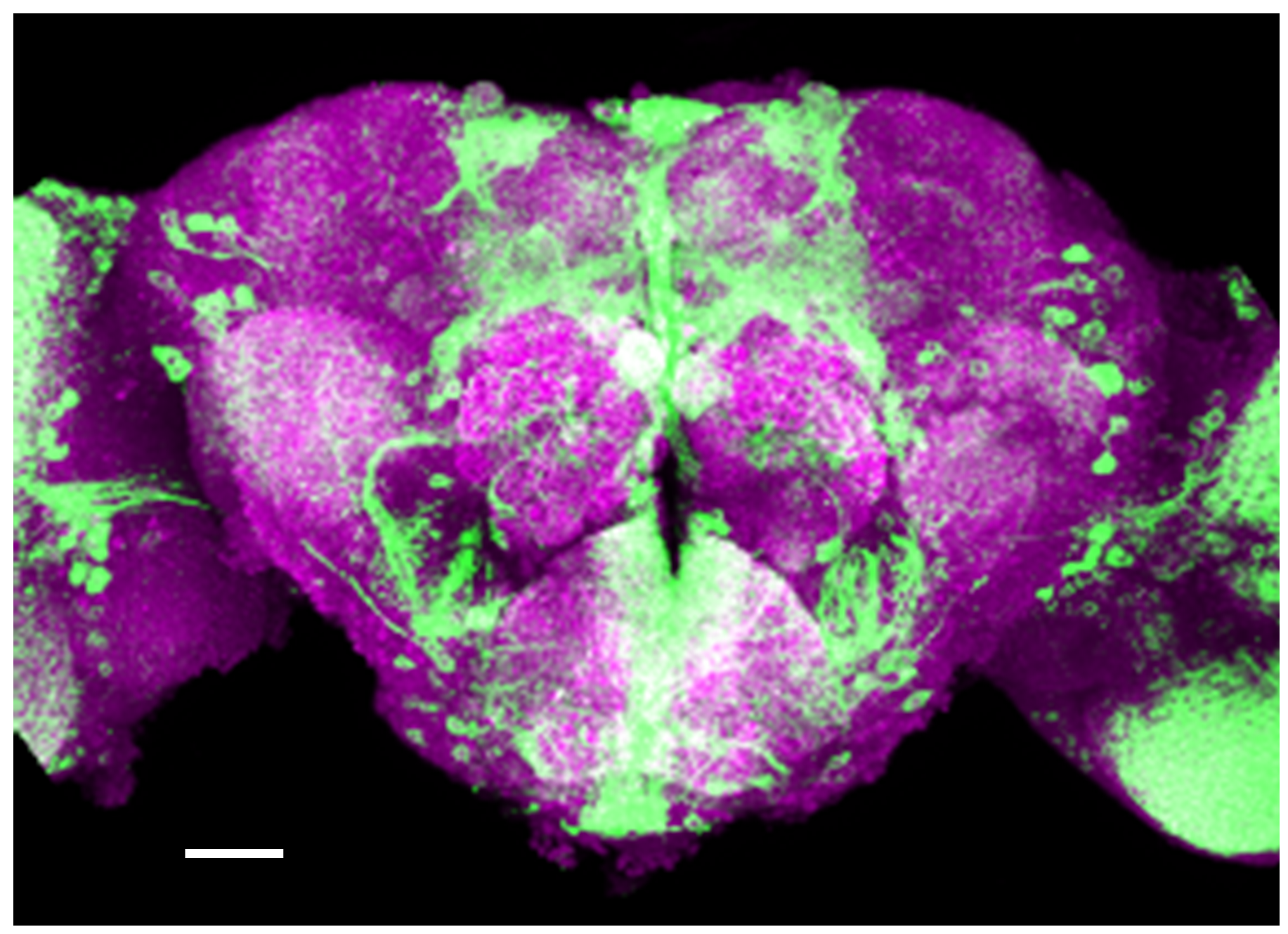

Supplement: S4 Fig — Scale bar, 20 μm. (TIF) [file pbio.1002586.s004.tif]
