# Supplementary material for: Suppression of Dopamine Neurons Mediates Reward
Source: PLoS Biol. 2016 Dec 20;14(12):e1002586. doi: 10.1371/journal.pbio.1002586 (PMC5172549; doi:10.1371/journal.pbio.1002586)
Supplement: S1 Table — (DOCX) [file pbio.1002586.s005.docx]

**S1 Table:** List of crosses and statistics for behavior experiments

| Figure | Group code | Males | Females | *n* | *D'Agostino & Pearson omnibus normality test*  *(P value)* | *Bartlett's test*  *(P value)* | *Bonferroni's (Dunn’s)*  *multiple comparisons test*  *(Adjusted P value)* |
| --- | --- | --- | --- | --- | --- | --- | --- |
| Figure 2A | *1* | *w; MB195B-GAL4* | *w* | *10* | *0.33* | *0.71* | *0.0002 (1 vs. 2) ****  *0.0052 (2 vs. 5) ***  *0.0009 (3 vs. 4) ****  *0.0022 (4 vs. 5) *** |
|  | *2* | *w; MB195B-GAL4* | *w; UAS-dTrpA1* | *9* | *0.74* |  |  |
|  | *3* | *w; MB441B-GAL4* | *w* | *8* | *0.79* |  |  |
|  | *4* | *w; MB441B-GAL4* | *w; UAS-dTrpA1* | *8* | *0.43* |  |  |
|  | *5* | *w* | *w; UAS-dTrpA1* | *7* | *NA* |  |  |
| Figure 2B | *1* | *w; MB195B-GAL4* | *w* | *8* | *0.001 *** | *NA* | *> 0.9999 (1 vs. 2)*  *0.1522 (2 vs. 5)*  *> 0.9999 (3 vs. 4)*  *0.0077 (4 vs. 5) ***  *(Dunn’s)* |
|  | *2* | *w; MB195B-GAL4* | *w; UAS-Shi^ts1^* | *8* | *0.61* |  |  |
|  | *3* | *w; MB441B-GAL4* | *w* | *8* | *0.46* |  |  |
|  | *4* | *w; MB441B-GAL4* | *w; UAS-Shi^ts1^* | *8* | *0.76* |  |  |
|  | *5* | *w* | *w; UAS-Shi^ts1^* | *8* | *0.68* |  |  |
| Figure 2C | *1* | *w; MB195B-GAL4* | *w* | *12* | *0.69* | *0.09* | *0.028 (1 vs. 2) **  *< 0.0001 (2 vs. 5) ****  *0.018 (3 vs. 4) **  *0.0083 (4 vs. 5) *** |
|  | *2* | *w; MB195B-GAL4* | *w; UAS-Shi^ts1^* | *12* | *0.64* |  |  |
|  | *3* | *w; MB441B-GAL4* | *w* | *18* | *0.81* |  |  |
|  | *4* | *w; MB441B-GAL4* | *w; UAS-Shi^ts1^* | *18* | *0.19* |  |  |
|  | *5* | *w* | *w; UAS-Shi^ts1^* | *20* | *0.79* |  |  |
| Figure 2D | *1* | *w; MB441B-GAL4* | *w* | *35* | *0.90* | *0.90* | *0.0083 (1 vs. 2) ***  *0.0028 (2 vs. 3) *** |
|  | *2* | *w; MB441B-GAL4* | *w; UAS-Shi^ts1^* | *34* | *0.77* |  |  |
|  | *3* | *w* | *w; UAS-Shi^ts1^* | *23* | *0.49* |  |  |
| Figure 2E | *1* | *w; MB195B-GAL4* | *w* | *8* | *0.24* | *0.16* | *0.644 (1 vs. 2)*  *0.981 (2 vs. 5)*  *> 0.9999 (3 vs. 4)*  *0.999 (4 vs. 5)* |
|  | *2* | *w; MB195B-GAL4* | *w; UAS-Shi^ts1^* | *8* | *0.51* |  |  |
|  | *3* | *w; MB441B-GAL4* | *w* | *8* | *0.66* |  |  |
|  | *4* | *w; MB441B-GAL4* | *w; UAS-Shi^ts1^* | *8* | *0.81* |  |  |
|  | *5* | *w* | *w; UAS-Shi^ts1^* | *8* | *0.38* |  |  |
| Figure 2F | *1* | *w; MB195B-GAL4* | *w* | *7* | *NA* | *NA* | *> 0.9999 (1 vs. 2)*  *0.999 (2 vs. 5)*  *> 0.9999 (3 vs. 4)*  *> 0.9999 (4 vs. 5)*  *(Dunn’s)* |
|  | *2* | *w; MB195B-GAL4* | *w; UAS-Shi^ts1^* | *7* | *NA* |  |  |
|  | *3* | *w; MB441B-GAL4* | *w* | *6* | *NA* |  |  |
|  | *4* | *w; MB441B-GAL4* | *w; UAS-Shi^ts1^* | *8* | *0.09* |  |  |
|  | *5* | *w* | *w; UAS-Shi^ts1^* | *8* | *0.24* |  |  |
| Figure 4B | *1* | *w; MB441B-GAL4* | *w* | *12* | *0.58* | *0.44* | *0.0013 (1 vs. 2) ***  *0.0021 (2 vs. 3) *** |
|  | *2* | *w; MB441B-GAL4* | *w; UAS-Shi^ts1^* | *11* | *0.67* |  |  |
|  | *3* | *w* | *w; UAS-Shi^ts1^* | *19* | *0.74* |  |  |
| Figure 4D | *1* | *w; MB441B-GAL4* | *w* | *8* | *0.74* | *0.26* | *0.019 (1 vs. 2) **  *0.0029 (2 vs. 3) *** |
|  | *2* | *w; MB441B-GAL4* | *w; UAS-eNpHR3* | *11* | *0.71* |  |  |
|  | *3* | *w* | *w; UAS-eNpHR3* | *9* | *0.67* |  |  |
| Figure 6A | *1* | *w;; AstA-GAL4* | *w* | *16* | *0.97* | *0.91* | *0.0146 (1 vs. 2) **  *0.0064 (2 vs. 3) *** |
|  | *2* | *w;; AstA-GAL4* | *w; UAS-dTrpA1* | *16* | *0.76* |  |  |
|  | *3* | *w* | *w; UAS-dTrpA1* | *16* | *0.94* |  |  |
| Figure 6B | *1* | *w; AstA-GAL4* | *w* | *8* | *0.67* | *0.83* | *0.0022 (1 vs. 2) ***  *0.0025 (2 vs. 3) *** |
|  | *2* | *w; AstA-GAL4* | *w; UAS-Shi^ts1^* | *8* | *0.64* |  |  |
|  | *3* | *w* | *w; UAS-Shi^ts1^* | *8* | *0.53* |  |  |
| Figure 6C | *1* | *w; AstA-GAL4* | *w* | *6* | *NA* | *NA* | *> 0.9999 (1 vs. 2)*  *> 0.9999 (2 vs. 3)*  *(Dunn’s)* |
|  | *2* | *w; AstA-GAL4* | *w; UAS-Shi^ts1^* | *8* | *0.69* |  |  |
|  | *3* | *w* | *w; UAS-Shi^ts1^* | *7* | *NA* |  |  |
| Figure 6D | *1* | *w; AstA-GAL4* | *w* | *6* | *NA* | *NA* | *0.690 (1 vs. 2)*  *> 0.9999 (2 vs. 3)*  *(Dunn’s)* |
|  | *2* | *w; AstA-GAL4* | *w; UAS-Shi^ts1^* | *8* | *0.17* |  |  |
|  | *3* | *w* | *w; UAS-Shi^ts1^* | *8* | *0.77* |  |  |
| Figure 6E | *1* | *w; AstA-GAL4* | *w* | *16* | *0.79* | *0.91* | *0.008 (1 vs. 2) ***  *0.036 (2 vs. 3) ** |
|  | *2* | *w; AstA-GAL4* | *w; UAS-Shi^ts1^* | *15* | *0.98* |  |  |
|  | *3* | *w* | *w; UAS-Shi^ts1^* | *9* | *0.30* |  |  |
| Figure 7A | *1* | *+* | *+* | *32* | *0.62* | *0.64* | *< 0.0001 (1 vs. 2) ****  *< 0.0001 (1 vs. 3) ****  *0.042 (1 vs. 4) ** |
|  | *2* | *w; AstA SK1* | *w; AstA SK1* | *22* | *0.76* |  |  |
|  | *3* | *w; AstA SK4* | *w; AstA SK4* | *20* | *0.60* |  |  |
|  | *4* | *w; AstA SK1* | *w; AstA SK4* | *10* | *0.35* |  |  |
| Figure 7B | *1* | *+* | *+* | *16* | *0.012 ** | *NA* | *> 0.9999 (1 vs. 2)*  *0.585 (1 vs. 3)*  *> 0.9999 (1 vs. 4)*  *(Dunn’s)* |
|  | *2* | *w; AstA SK1* | *w; AstA SK1* | *8* | *0.50* |  |  |
|  | *3* | *w; AstA SK4* | *w; AstA SK4* | *8* | *0.69* |  |  |
|  | *4* | *w; AstA SK1* | *w; AstA SK4* | *8* | *0.23* |  |  |
| Figure 7C | *1* | *w; AstA-GAL4* | *+* | *12* | *0.26* | *0.65* | *0.0046 (1 vs. 2) ***  *0.034 (2 vs. 3) ** |
|  | *2* | *w; AstA-GAL4* | *+; UAS-AstA-RNAi* | *16* | *0.28* |  |  |
|  | *3* | *w* | *+; UAS-AstA-RNAi* | *16* | *0.35* |  |  |
| Figure 7D | *1* | *w; AstA-GAL4* | *+* | *8* | *0.99* | *0.54* | *0.706 (1 vs. 2)*  *0.9923 (2 vs. 3)* |
|  | *2* | *w; AstA-GAL4* | *+; UAS-AstA-RNAi* | *8* | *0.52* |  |  |
|  | *3* | *w* | *+; UAS-AstA-RNAi* | *8* | *0.28* |  |  |
| Figure 9A | *1* | *w; MB441B-GAL4* | *+* | *15* | *0.67* | *0.004 *** | *0.0021 (1 vs. 2) ***  *0.0042 (2 vs. 3) ***  *(Dunn’s)* |
|  | *2* | *w; MB441B-GAL4* | *+; UAS-DAR-1-RNAi* | *12* | *0.64* |  |  |
|  | *3* | *w* | *+; UAS-DAR-1-RNAi* | *15* | *0.53* |  |  |
| Figure 9B | *1* | *w; MB441B-GAL4* | *+* | *25* | *0.11* | *0.08* | *0.042 (1 vs. 2) **  *0.019 (2 vs. 3) ** |
|  | *2* | *w; MB441B-GAL4* | *+; UAS-DAR-1-RNAi-2* | *24* | *0.58* |  |  |
|  | *3* | *w* | *+; UAS-DAR-1-RNAi-2* | *26* | *0.64* |  |  |
| Figure 9C | *1* | *w; MB441B-GAL4* | *+* | *9* | *0.91* | *0.61* | *0.0941 (1 vs. 2)*  *0.292 (2 vs. 3)* |
|  | *2* | *w; MB441B-GAL4* | *+; UAS-DAR-1-RNAi* | *9* | *0.48* |  |  |
|  | *3* | *w* | *+; UAS-DAR-1-RNAi* | *9* | *0.96* |  |  |
| Figure 9D | *1* | *w; MB441B-GAL4* | *+* | *8* | *0.23* | *0.24* | *0.946 (1 vs. 2)*  *0.428 (2 vs. 3)* |
|  | *2* | *w; MB441B-GAL4* | *+; UAS-DAR-1-RNAi-2* | *8* | *0.14* |  |  |
|  | *3* | *w* | *+; UAS-DAR-1-RNAi-2* | *8* | *0.87* |  |  |
| Figure 10A | *1* | *+* | *w; MB441B-GAL4* | *8* | *0.71* | *0.15* | *0.038 (1 vs. 2) **  *0.034(2 vs. 3) ** |
|  | *2* | *+; UAS-PTX16* | *w; MB441B-GAL4* | *8* | *0.76* |  |  |
|  | *3* | *+; UAS-PTX16* | *w* | *8* | *0.34* |  |  |
| Figure 10B | *1* | *+* | *w; MB441B-GAL4* | *8* | *0.57* | *0.70* | *0.909 (1 vs. 2)*  *0.982 (2 vs. 3)* |
|  | *2* | *+; UAS-PTX16* | *w; MB441B-GAL4* | *8* | *0.61* |  |  |
|  | *3* | *+; UAS-PTX16* | *w* | *8* | *0.53* |  |  |
| Figure S1 | *1* | *w; R58E02-GAL4* | *w* | *16* | *0.16* | *0.19* | *> 0.9999 (1 vs. 2)*  *> 0.9999 (2 vs. 3)*  *(Dunn’s)* |
|  | *2* | *w; R58E02-GAL4* | *w; UAS-Shi^ts1^* | *16* | *0.04 ** |  |  |
|  | *3* | *w* | *w; UAS-Shi^ts1^* | *16* | *0.42* |  |  |
| Figure S2 | *1* | *w; MB441B-GAL4* | *w* | *10* | *0.43* | *NA* | *0.048 (1 vs. 2) **  *0.032 (2 vs. 3) **  *(Dunn’s)* |
|  | *2* | *w; MB441B-GAL4* | *w; UAS-Shi^ts1^* | *8* | *0.01 ** |  |  |
|  | *3* | *w* | *w; UAS-Shi^ts1^* | *10* | *0.71* |  |  |
| Figure S3A | *1* | *w; MB441B-GAL4* | *w* | *8* | *0.64* | *0.34* | *0.242(1 vs. 2)*  *0.973 (2 vs. 3)* |
|  | *2* | *w; MB441B-GAL4* | *w; UAS-Shi^ts1^* | *8* | *0.64* |  |  |
|  | *3* | *w* | *w; UAS-Shi^ts1^* | *8* | *0.80* |  |  |
| Figure S3B | *1* | *w; AstA-GAL4* | *w* | *8* | *0.78* | *0.26* | *0.020 (1 vs. 2) **  *0.940 (2 vs. 3)* |
|  | *2* | *w; AstA-GAL4* | *w; UAS-Shi^ts1^* | *8* | *0.63* |  |  |
|  | *3* | *w* | *w; UAS-Shi^ts1^* | *8* | *0.80* |  |  |
